# Uptake of Large Language Models by London Medical Students: Exploratory Qualitative Interview Study

**DOI:** 10.2196/82828

**Published:** 2026-01-19

**Authors:** Maya Alazzawi, Kyle Lam

**Affiliations:** 1 Department of Surgery and Cancer Imperial College London London United Kingdom

**Keywords:** large language models, artificial intelligence, AI, generative artificial intelligence, undergraduate medical education

## Abstract

**Background:**

The popularity of large language models (LLMs) has grown exponentially across health care. Despite the wealth of literature on proposed applications in medical education, there remains a critical gap regarding their real-world use, benefits, and challenges as experienced by medical students themselves.

**Objective:**

We aimed to explore qualitatively and characterize the perceived benefits, facilitators, and barriers associated with the use of LLMs among a cohort of London-based medical students.

**Methods:**

Semistructured interviews were conducted with 15 medical students from preclinical and clinical stages at London-based medical schools. Guided by the technology acceptance model, interview transcripts underwent an inductive thematic analysis to identify themes on actual system use, perceived usefulness, ease of use, and attitudes toward LLMs.

**Results:**

All participants reported frequent use of ChatGPT for concise topic summarization, clarification of complex concepts, generation of examination-style questions, and summarization of research. Students described LLMs as a complementary tool to traditional materials, valuing their immediacy (“Instead of getting a textbook, I can ask ChatGPT to summarise something in X words and read it in under a minute”) and ease of use. Peer demonstration and device-agnostic accessibility emerged as key facilitators. Of note, wider applications such as simulating clinical interviews were discovered through peers rather than through formal teaching. Significant barriers were reported. Hallucinations, fabricated references, and outdated information led to loss of trust, with more junior students finding inaccurate outputs difficult to detect (“I stopped using it because I found it to be inaccurate, and I don’t want to be learning the wrong things”). Half of the participants interviewed reported a sense of overreliance, defaulting to its use for answers with a perceived loss of critical thinking ability. Students noted inequalities in access to advanced features and voiced concerns about privacy when using LLMs in clinical scenarios.

**Conclusions:**

LLMs have been widely adopted by medical students. While students perceived the efficiency, flexibility, and conversational interface of LLMs as beneficial, substantial reservations remain regarding their reliability, potential de-skilling, and the loss of academic integrity. These findings underpin the urgent need for curricula to both support safe LLM use and also adapt assessment and teaching strategies for artificial intelligence–augmented student practice. Future research should broaden geographical representation, investigate applications in low-resource settings, and integrate educators’ perspectives to establish future curricular guidance in an artificial intelligence era.

## Introduction

Artificial intelligence (AI) has become increasingly prevalent in the modern world. In November 2022, the release of ChatGPT by OpenAI marked a pivotal moment in the evolution of large language models (LLMs). Its success was not solely due to demonstrating exceptional generative abilities and maintaining humanlike conversational interactions but arguably even more significantly due to bridging the technological divide and making this advanced technology accessible to the public. Specifically in health care, LLMs have been demonstrated to encode significant clinical knowledge [1], and potential uses within clinical care include their use in emergency department triage, automation of clinical documentation [2], and patient chatbots [3].

While these use cases have been hypothesized to have significant clinical impact, the risks of implementing LLMs in real-world clinical practice are significant. Hallucinations, where LLMs output plausible but incorrect responses, could have profound consequences for patient safety, and existing health technology regulations remain behind the curve with these dynamic systems that do not have a singular defined use case. Medical education represents a potentially high-impact but low-risk application and has been no exception to the growing influence of LLMs. LLMs have been widely publicized to be able to pass both undergraduate and postgraduate medical examinations [4,5]. While impressive, the real benefits of these models lie in how users can use them to gain knowledge more readily rather than in the information encoded within these models themselves. The existing literature surrounding LLMs has proposed multiple use cases for medical education, including generation of topic explanations and summaries [6], simulated and interactive physician-patient interactions [7], and generating high-quality examination questions [8].

Despite these advances, there remains a significant gap in the development of comprehensive curriculum frameworks for AI and LLMs in undergraduate medicine, and reviews have found heterogeneity in learning objectives and evaluation methods and limited standardization across programs [9,10]. Against a backdrop of rapid uptake of LLMs by students and clinicians, this lack of structured education and guidance creates an immediate skill gap for safe and effective use of LLMs in training and practice. Existing cross‑sectional surveys report that many medical students are already experimenting with generative AI while receiving minimal structured training or guidance, highlighting a gap between adoption and pedagogy [11,12]. One survey of US medical students, for example, found that only 8.8% had received any resources to explore AI in medicine despite over 90% agreeing that its inclusion in the curriculum would benefit their future career [13]. Multiple institutional and regional surveys similarly show high familiarity and growing use but inconsistent formal teaching, suggesting that independent student use has outpaced curriculum development and assessment [14]. Analyses of curriculum frameworks and programs have concluded on the need for clearer delivery models and assessment strategies to evaluate skills such as appraisal of AI outputs, safe prompt practices, and documentation of learning outcomes [10]. These surveys show that students and clinicians report positive attitudes toward integrating AI although they have concerns about ethics and reliability with use cases, including writing support, exam preparation, and quick explanations rather than structured learning pathways [11].

While these often single-institutional cross-sectional studies do capture valuable insights, they are often based on self-reported data and rarely probe into real-world practice, such as verification behaviors and how uncertainty is handled. To address these gaps in the literature, we conducted a qualitative interview study among medical students at London medical schools to complement the existing breadth of the survey literature with depth on actual practices. Therefore, this survey explored, first, how LLMs are currently used in real-world educational settings; second, the perceived benefits of, barriers to, and facilitators of adoption; and, finally, the implications for future curriculum and policy design. We aimed to report findings that will provide valuable insights for educators and regulators to allow for the safe and effective integration of LLMs into undergraduate medical training.

## Methods

### Study Design and Rationale

We selected semistructured interviews for data collection as they allowed for in-depth exploration of participants’ attitudes, beliefs, and behaviors regarding LLM use. These are objectives not easily achieved using structured interviews. The flexibility of the semistructured format enabled the interviewer to probe participant responses, clarify ambiguities, and follow lines of inquiry, therefore allowing for richer and more context-dependent responses. The semistructured interview approach is particularly well suited to exploring complex or emerging phenomena, such as the adoption of novel technologies in education, and is endorsed by qualitative research best practices for trustworthiness and depth [15]. A limitation of this approach is the potential for reduced consistency across interviews and increased reliance on interviewer skill, which we mitigated through a standardized topic guide (Multimedia Appendix 1).

### Interview Topic Guide Development

**Figure 1:**
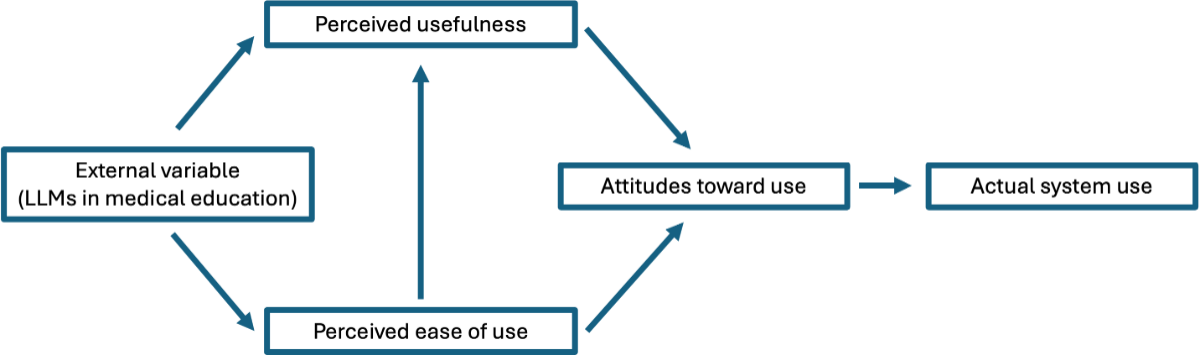
Components of the technology acceptance model (TAM) and relationship between variables. LLM: large language model.

The standardized topic guide was created based on themes identified from the current literature regarding the use of LLMs and medical education. This was structured around the technology acceptance model (TAM), a widely applied model of user acceptance and use of technology [16]. The TAM is structured around the perceived ease of use and perceived usefulness of a new technology, which describe the effect of these on attitude toward using and actual system use of the technology (Figure 1). By structuring the topic guide around the TAM, the questions focused on specific use cases of LLMs within medical education, their perceived usefulness in medical education, and the ease of use and attitudes toward use.

### Participant Recruitment and Sampling

Recruitment was conducted through open advertising to all London medical students via university mailing lists and posters (Multimedia Appendix 1). Any medical student at any stage of training was eligible for inclusion, with no strict exclusion criteria. As such, this approach constituted convenience sampling. No sample size was set a priori in line with other qualitative studies. Interviews were conducted until thematic saturation was achieved (no new prominent themes were observed).

### Data Collection

All interviews were conducted by one female researcher (MA) over Microsoft Teams, where they were also recorded and transcribed. The lead interviewer (MA) is both a medical student and a researcher with an interest in LLMs and digital education. While this enabled her to understand the context of narratives, this also introduced the potential for shared biases (eg, assumptions about the value or risks of LLM adoption). To mitigate these risks, the interviewer adhered to a prespecified semistructured interview guide, taking field notes before and after interviews, and regularly discussed analytic decisions and coding with a coresearcher (KL) who also independently validated the codes. Participants were not known to the interviewer. The interviewer introduced the study and the rationale for the study to the participants. The interviewer also intentionally adopted a neutral stance, used open-ended questioning, and explicitly invited participants to discuss negative experiences and skepticism regarding LLMs. This approach aimed to ensure that a full range of perspectives were actively solicited and represented in the final analysis.

### Data Analysis

Data were analyzed using an inductive thematic analysis methodology guided by the TAM as a deductive thematic framework. The analytic approach began with repeated reading of transcripts and listening back to recordings to ensure immersion and familiarization with the data while noting initial impressions. The first author (MA) then undertook line-by-line open coding of all transcripts, assigning short, descriptive labels to segments of text to capture participants’ reported behaviors and perceptions and experiences of LLM use without imposing preexisting categories. Codes were then iteratively refined, grouped, and compared across transcripts and organized into candidate themes. These themes were subsequently mapped onto domains of the TAM (system use, ease of use, perceived usefulness, and attitudes toward use), with the codebook and thematic structure independently checked and refined by a second researcher (KL; Multimedia Appendix 1).

### Ethical Considerations

As a low-risk undergraduate project, this study was deemed exempt from ethics approval by the research ethics committee at Imperial College London and was approved at the departmental level [17]. Therefore, the project was not formally reviewed by an ethics committee but was reviewed and approved by the head of department. All participants gave informed consent before taking part in the study. All interview transcripts and subsequent analyses were anonymized to safeguard participant information. Participants were not compensated for taking part.

## Results

### Overview

A total of 15 medical students from all years were interviewed across 2 London medical schools, comprising 7 (46.7%) preclinical students and 8 (53.3%) clinical students (Table 1), with an average interview length of 19.4 (SD 4.2) minutes. Thematic analysis was mapped to the TAM, and each subtheme was mapped to the study aims of benefit, barrier, or facilitator. Table 2 provides frequencies and illustrative quotes. All proportions and percentages presented are based on our qualitative, nonrandom sample of 15 students and serve as descriptive indicators of this specific group. No statistical inference or generalization beyond the study population was intended, and the findings should be interpreted as illustrative of this cohort rather than representative of the broader medical student population.

Despite the availability of other LLMs, all interviewees spontaneously described the use of ChatGPT, with none routinely using alternative LLMs. As such, our results focus primarily on ChatGPT as the representative LLM. This is reflective of the dominant adoption pattern in UK medical education at the time of data collection in line with surveys in the literature.

**Table 1:** Demographic characteristics of the participants (N=15).

Demographic and category			Values
Age (y), mean (SD; range)			20.3 (1.4; 18-23)
Stage of study, n (%)			
	Preclinical	7 (46.7)	
	Clinical	8 (53.3)	
Sex, n (%)			
	Female	11 (73.3)	
	Male	4 (26.7)	
Race or ethnicity, n (%)			
	Arab	4 (26.7)	
	Asian	6 (40)	
	White	5 (33.3)	

**Table 2:** Thematic analysis of interviews with themes based on the structure of the technology acceptance model (TAM) and subthemes mapped to benefits, facilitators, and barriers with example quotes (N=15).

TAM theme and subtheme			Prevalence, n (%)		Aim mapping		Example quote
Actual system use							
	Topic summarization	15 (100)		Benefit		“Instead of getting a textbook, I can ask ChatGPT to summarise something for me in X words and read it under a minute or two.”	
	Google or search engine replacement	13 (86.7)		Benefit		“It’s just better than Googling it.”	
	Quiz or exam question creation	6 (40)		Benefit and facilitator		“I give it a topic...and say ‘make some test questions,’ and I specify the difficulty and style as well.”	
	History taking role-play	5 (33.3)		Benefit and facilitator		“It’s a nice environment to test out sentences or questions before a real patient.”	
	Email drafting or administrative tasks	3 (20)		Benefit		“I’ve used it for writing emails and admin tasks like that.”	
	Article or research summary	7 (46.7)		Benefit		“It enabled me to spend less time reading tons of papers to find an answer.”	
Perceived usefulness							
	Efficiency and time saving	13 (86.7)		Benefit		“It’s more of a natural conversation as opposed to Google, where you have to scroll through lots of sites.”	
	Clarifying difficult concepts	5 (33.3)		Benefit		“If there’s a difficult concept, I ask ChatGPT to summarise it in simple terms.”	
	Mnemonic or flash card generation	3 (20)		Benefit		“It can come up with mnemonics and memory aids—though they’re not always very good.”	
	Change in educational assessment needs	10 (66.7)		Facilitator and barrier		“If everyone is using ChatGPT to study, assessments may need to be adjusted...”	
Perceived ease of use							
	Device flexibility	6 (40)		Facilitator		“It fits into my revision routine because I can use it on my phone in clinic.”	
	Technical simplicity and low learning curve	7 (46.7)		Facilitator		“You just type your question and it gives you what you want.”	
Attitudes toward use							
	Overreliance	8 (53.3)		Barrier		“It’s almost like an addiction...outsourcing every little bit of thinking instead of working something out.”	
	Collaborative learning via peer demonstration	9 (60)		Facilitator		“After my friend showed me, I started using it for new things.”	
	Lack of awareness of large language model features	8 (53.3)		Barrier and facilitator		“That would be useful, but I’ve never used it for that before.”	
	Hallucinations or inaccurate answers	15 (100)		Barrier		“I stopped using it...because I found it to be inaccurate, and I don’t want to be learning the wrong things.”	
	Generation of fake or nonexistent references	8 (53.3)		Barrier		“It almost makes up sources out of thin air. You paste the reference in a browser, and it doesn’t exist.”	
	Difficulty trusting without prior knowledge	9 (60)		Barrier		“I think you need to have a bit of an understanding already to make sure what you’re being told is right.”	
	Privacy and data concerns	5 (33.3)		Barrier		“I’m wary of using ChatGPT in research because I don’t understand all the copyright implications.”	
	Out-of-context output	6 (40)		Barrier		“Sometimes the information is just out of context, so you have to clarify...get more specific with prompts.”	
	Preference for official or older resources	7 (46.7)		Barrier		“I have access to sources which are more reliable than ChatGPT, like older years’ notes or textbooks.”	
	Inadequate for guideline or recommendation queries	9 (60)		Barrier		“I don’t think I’d use it for treatment guidelines. Easier to get it from NICE or textbooks.”	

### Perceived Benefits

Students consistently described how ChatGPT had changed their learning practices, accelerated knowledge acquisition, and improved their study efficiency. Use cases reported by participants included use of ChatGPT for concise topic summarization, clarification of complex concepts, and supplementing traditional learning materials such as textbooks. ChatGPT was reported by participants as complementary to textbooks and peer notes, allowing students to obtain information on demand, with one student reporting the following:

Instead of getting a textbook, I can ask ChatGPT to summarise something in X words and read it in under a minute.

Wider uses of ChatGPT within a medical education context included 40% (6/15) of the students using the technology to generate replica exam-style questions and immediate answer explanations, with students reporting its value for reinforcing knowledge and identifying gaps. A total of 20% (3/15) of the students asked ChatGPT to create mnemonics, flash cards, or analogies to aid memorization, although the quality of system outputs was acknowledged to be variable. Almost half (7/15, 46.7%) of the participants reported that ChatGPT was integrated within their learning workflow particularly in research and essay preparation as it could provide summaries of academic papers and structure long-form writing:

It enabled me to spend less time reading tons of papers to find an answer.

Participants also reported turning to ChatGPT when traditional search engines failed to provide adequate answers. In total, 33.3% (5/15) of the students reported that ChatGPT provided answers for academic queries that they felt that Google or standard resources were unable to solve. Within clinical education settings, 33.3% (5/15) of the students described using ChatGPT to simulate history taking and patient role-play scenarios. They valued its ability to offer feedback on question phrasing and clinical reasoning before performing these tasks on real patients. Of note, participants did not trust LLMs to output appropriate treatment guidelines and consistently expressed a preference for official sources such as the National Institute for Health and Care Excellence. Finally, beyond allowing for gains in efficiency, participants reported that the conversational interface of ChatGPT allowed them to clarify concepts in real time, supporting a shift from pure factual recall toward inquisitive, analytical learning.

### Facilitators

A key facilitator identified by participants was peer demonstration and collaborative exploration. Over half (9/15, 60%) of the students were introduced to use cases and wider applications through friends (“After my friend showed me, I started using it for new things”). The ease of using the platform combined with device flexibility (ie, ChatGPT can be used across laptops, desktop computers, and smartphones) was also identified as a key facilitator to widespread adoption and allowed it to be integrated into study routines regardless of location (“It fits into my revision routine because I can use it on my phone in clinic”).

For students who were anxious about live clinical encounters, the ability to simulate history-taking scenarios was a valued educational facilitator. Participants commented that the safe and unpressed environment of ChatGPT was beneficial for rehearsing questioning techniques and receiving unbiased feedback, allowing them to develop their skillsets. Students also commented positively on how the tool was instantly accessible, the lack of institutional gatekeeping, and the lack of learning curve to use.

However, students did broadly acknowledge that the widespread use of LLMs among students would mean that assessment strategies would have to be reformed and that examination styles would have to shift toward more critical thinking approaches.

### Barriers

While broadly there was enthusiasm toward ChatGPT’s utility among participants, there were significant reservations regarding its reliability. All interviewees were able to recount experiences in which outputs were either outdated or hallucinated. Many noted that ChatGPT often seemed plausible when incorrect, which made detecting errors challenging when students lacked deeper subject knowledge. This led to students avoiding the use of ChatGPT when trying to grasp the foundations of a topic, with one student explaining the following:

I stopped using it…because I found it to be inaccurate, and I don’t want to be learning the wrong things.

A total of 53.3% (8/15) of the students highlighted how ChatGPT hallucinated research references, encountering fabricated citations that could not be traced to real sources. Therefore, this led to participants perceiving the need for rigorous manual verification when using ChatGPT for academic projects, recognizing its inability to perform critical appraisal typical of research:

LLMs skip through this critical appraisal when giving you the information.

Themes of overreliance and cognitive outsourcing also emerged among students, with participants describing an increasing tendency to default to ChatGPT for problem-solving and academic tasks that previously required more effort and perceiving that this could risk the erosion of traditional skills. Some students even felt a self-perceived addiction, with a fear that the ability to instantly receive answers could diminish perseverance and engagement with more complex material.

Other barriers noted included frustration with out-of-context outputs, requiring multiple prompts to obtain clinically usable or relevant information. Several participants also cited privacy and data concerns, with anxieties about copyright implications and uncertainty regarding data security. Awareness of broader functionalities of ChatGPT was uneven. While some students were simply unaware that ChatGPT could have broader applications such as custom question generation, others deliberately limited use out of concern for output reliability.

## Discussion

### Key Findings in Context

This qualitative study is, to our knowledge, the first to explore how medical students are adopting, using, and perceiving LLMs for education using in-depth semistructured interviews. By mapping the TAM to user behaviors, this analysis offers an account of both the facilitators of and barriers to real-world LLM adoption in undergraduate medicine. This work contributes a qualitative account of how and why medical students are using LLMs, mapping not only the applications of LLMs within medical education but also the drivers of their uptake.

Our study shows the widespread and largely self-directed uptake of LLMs among medical students. There is an emerging shift toward these tools, replacing long-standing educational tools, including web searches or traditional textbooks, in favor of LLMs due to their capabilities of rapid summarization; conversational clarification; and device-agnostic, on-demand engagement. This signals a shift within medical education toward AI-augmented learning in which efficient, personalized, and readily accessible tools are perceived as an improvement over traditional materials.

While many of the proposed use cases for LLMs in medical education have been reported in the literature, our findings suggest that most students have not adopted the full spectrum of capabilities reported by conceptual work. Instead, real-world adoption remains focused on a relatively narrow set of functions. One survey of 443 medical students found that “a sizeable portion of students lack knowledge about ChatGPT’s various functions and limitations” [18]. Complementary analyses in the literature have also highlighted a critical distinction between technology adoption and technological literacy. While institutions are rapidly introducing AI tools, medical curricula have lagged in providing structured training for responsible use, creating a gap between tool uptake and competency development [19]. Digital health frameworks have also emphasized that competence with tools such as LLMs should be developed longitudinally across the curriculum rather than acquired informally through peer networks [20]. Our observation that students discovered advanced use cases predominantly through peers rather than formal teaching demonstrates this pedagogical gap and highlights the need for AI literacy training at an undergraduate level.

Such a gap poses significant problems. First, there is an opportunity gap in which students may lack the awareness of how LLMs can be widely used. Second, and perhaps more importantly, students may not be aware of the limitations of LLMs, trusting potentially inaccurate outputs that could be applied in a clinical setting. Efforts to solve these issues have already been made with the recent publication of the digital health competencies in medical education framework [21]. While this is a vital first step to grapple with the challenges previously discussed, generative AI continues to evolve on a weekly basis with growing capabilities, and educational leaders must also decide how to ensure that the education of students does not lag behind the innovation curve.

We also demonstrated that enthusiasm about LLMs is also met with concerns. Hallucinations, nonexistent references, and out-of-date information were reported by all participants, with comments including “I stopped using it to help give answers to questions because I found it to be inaccurate, and I don’t want to be learning the wrong things” and “It does a very good job of making it seem like everything is conducive to each other, but sometimes it says things that were probably true at one point but aren’t true anymore.” These have been widely documented as critical limitations in the literature [22,23]. These reliability concerns are especially critical for more junior students who may lack the knowledge to critically assess outputs and are most at risk of “accepting what ChatGPT tells you” at face value. Recent work has validated these students’ experiences. When ChatGPT was prompted to generate academic references, approximately 1 in 5 citations was entirely fabricated, and over half of all citations contained at least one substantive error [24]. Strategies to mitigate LLM hallucinations, such as via prompt engineering, have been found to be only partially successful, with some prompting strategies even increasing major errors [25,26]. Current LLMs may also lack mechanisms to signal uncertainty and, together with frequently fabricated citations, this limits users’ ability to appraise reliability at the point of use [27]. Therefore, such limitations demand that students possess at least a basic skillset in assessing AI outputs.

Finally, over half of the students interviewed acknowledged overreliance on LLMs, with both reduced incentives to use trusted sources and a feeling of reduced problem-solving abilities. We demonstrated that perceived overreliance and addiction to these tools, which have been previously theorized in the literature, may already exist in real-world practice [28]. Recent commentary has proposed potential mechanisms underpinning this pattern, including automation bias, cognitive off‑loading, and genuine de-skilling of foundational clinical and academic skills in novice learners. Proposed countermeasures include reform in assessment approaches and explicit requirements for learners to interrogate, justify, and potentially reject AI‑generated suggestions using primary sources [29].

### Uptake of LLMs Requires Training and Assessment Reform

Facilitators of effective LLM adoption included social and peer-led dynamics. Broader use cases of LLMs, including custom question generation and history taking simulation, were discovered informally through friends and peers rather than formally taught. This demonstrates that peer-led learning rather than formal curricula is driving LLM adoption. Although these patterns may drive adoption and innovation, they may also propagate unsafe or suboptimal practices in the absence of formal oversight. In the context of potential LLM use in clinical settings, there is an urgent need for formalized training to be implemented to address safe and responsible use.

Our findings demonstrate that students are aware that the growing uptake of LLMs will likely require reform in assessment strategies. Participants, in line with the literature, anticipated that assessments will move away from factual recall and toward higher-order reasoning, critical appraisal, and digital literacy [30]. Recent conceptual work has begun to propose strategies for such reform, including “process‑focused”grading that evaluates students explicitly assuming that AI tools may have been used rather than judging only the final answer [29]. Other proposals include “AI‑resistant” question formats and assessment models in which learners are presented with a mixture of accurate and flawed AI‑generated responses and required to accept, modify, or reject them with justification from primary evidence [19]. Embedding these approaches within competency‑based curricula could protect academic integrity while simultaneously cultivating the skills needed to work safely with LLMs.

### Ethical Considerations of LLM Use

Although LLMs were recognized to be readily accessible, the existence of the “freemium” model means that certain advanced features may only be available through paid versions of LLMs (such as ChatGPT Plus) or through institutional arrangements. This access gap has potential consequences surrounding fairness and opportunity and may drive a digital divide leading to inequalities in medical education whereby students may be disadvantaged as they cannot afford to pay for a premium LLM. This concern reflects broader evidence on digital inequity in health care education. A recent systematic review has highlighted that disparities in technology access represent a fundamental barrier to health equity, which particularly affects students from lower socioeconomic backgrounds [31]. Moreover, medical education that adopts AI without universal access mechanisms risks exacerbating existing inequalities and biases encoded in AI training data, which typically originate from high-income settings and underrepresent marginalized populations [32].

As LLM use becomes increasingly mainstream, particularly in clinical education, it also poses risks to patient privacy and confidentiality. Current regulatory frameworks provide general data protection guidance, but implementation strategies specific to generative AI remain underdeveloped [33]. When students input clinical details into commercial LLMs, they risk inadvertently introducing identifiable patient information into systems with unclear data governance.

Addressing the interconnected concerns of access, bias, and privacy requires that medical schools implement equity and privacy impact assessments before adopting LLMs, ensure institutional provision of secure access for all students, and develop curricula that ensure critical awareness of algorithmic bias and data governance alongside AI literacy.

### Limitations and Future Work

This study has several limitations. First, all participants were recruited from only 2 medical schools located in London, and therefore, the findings of this study should be interpreted as locally specific and exploratory. Due to the convenience sample and restriction to a single metropolitan area, our results cannot be generalized beyond the surveyed London cohort, and we acknowledge that claims of saturation may be limited to this cohort.

The sample may also be subject to selection bias, potentially overrepresenting students who are early adopters, more digitally engaged, or have a preexisting interest in LLMs and AI, particularly given the use of open advertising to recruit participants. Broader studies are required to establish the generality of these patterns in other geographic and curricular settings as geographical diversity could lead to different experiences, perceptions, and use patterns of LLMs.

Furthermore, the impacts and potential utility of LLMs in low-resource setting medical schools remain unexplored. Medical students in these environments may derive distinct benefits from LLMs due to differing digital infrastructures, educational resources, and availability of teaching staff [34]. Further work should also extend to investigate the application and acceptability of LLMs in international and lower-resource contexts to ascertain whether LLMs could effectively bridge educational disparities.

Finally, this study did not explore the perspectives of educators or curriculum developers, who play crucial roles in incorporating AI into medical education. Understanding educators’ and curriculum designers’ views on what should be included in AI-related medical education and how these competencies should be evaluated and continuously updated is critical.

Therefore, future research will engage these stakeholders to inform comprehensive curricular frameworks that ensure that students are adequately prepared for responsible and effective use of LLMs in their future clinical practice. Further work will also aim to assess longitudinal impacts of LLM use on educational outcomes, clinical reasoning skills, and competencies over time. Investigating longitudinal changes in students’ critical thinking and problem-solving capabilities may provide valuable insights into whether and how educational assessments should evolve to reflect the realities of LLM use. Finally, ethical considerations, including privacy, data protection, and academic integrity, should be taken into account, and pathways should be created to address differences in access and bridge digital divides.

### Conclusions

This study provides a qualitative exploration into the real-world use, perceived benefits, and barriers regarding LLMs among a cohort of London-based medical students. LLMs have been widely adopted by medical students largely due to their ease of use, conversational interactions, and efficiency compared to traditional educational resources. However, there is a significant disparity between real-world use cases and those proposed in the literature. Significant concerns also remain regarding reliability, accuracy, and the risk of overreliance on these tools, potentially impacting critical thinking and clinical decision-making skills. These findings underscore the urgent need for structured education surrounding AI itself, as well as the broader implications of AI technologies on medical education delivery, curriculum design, and assessment methods. LLMs are likely here to stay, and we should be responsive adapting ahead of this adoption curve. Therefore, future educational initiatives not only should focus on developing AI competencies but also must adapt assessments to prioritize higher-order skills such as evaluation, critical thinking, and clinical reasoning, ensuring that medical students remain proficient practitioners in an era increasingly shaped by generative AI. Further research is necessary to explore geographic diversity, implications in low-resource contexts, and educators’ perspectives to comprehensively inform curriculum development and ensure the effective integration of these technologies.

## Appendix

Multimedia Appendix 1 Topic guide, codebook, and recruitment poster.

## Abbreviations

AI: artificial intelligence
LLM: large language model
TAM: technology acceptance model

## Data Availability

The datasets generated or analyzed during this study are available from the corresponding author on reasonable request.
