# Supplementary material for: Uptake of Large Language Models by London Medical Students: Exploratory Qualitative Interview Study
Source: JMIR Form Res. 2026 Jan 19;10:e82828. doi: 10.2196/82828 (PMC12865347; doi:10.2196/82828)
Supplement: Multimedia Appendix 1 [file formative_v10i1e82828_app1.docx]

**Supplementary Material**

**Topic Guide**

|  | **Question** | **Domain of the TAM** |
| --- | --- | --- |
| **1** | What stage of your medical school are you at? |  |
| **2** | How have you normally studied for your medical school exams? |  |
| **3** | Would you be able to share your experiences and awareness of the use of LLMs in medical education? | Attitude towards use |
| **4** | Where have you used LLMs in your medical career? | Actual system use |
| **5** | What do you think the value of LLMs is in your studies?   - Value to understanding medical topics. - Value to creating flashcards for you. - Value to providing information on treatment guidelines. - Value to generating quizzes and providing feedback - Value to stimulating patient interaction - Value to assisting research - Are you able to share personal experience of the benefit of LLMs? | Perceived usefulness |
| **6** | What do you think are the potential disadvantages of using LLMs in medical education?   - Information contextualisation - Incorrect / out-of-date information - Lack of human understanding | Perceived usefulness |
| **7** | How easy or difficult do you find LLMs to use during your medical studies? | Perceived ease of use |
| **8** | What aspects of LLMs make it easy or difficult to understand when using for medical education? | Perceived ease of use |
| **9** | If you haven’t used LLMs in your medical education, what are the reasons for this? | Attitude towards use |
| **10** | Has there been a time where you have used LLMs and it has not been accurate or the results you received made you less inclined to use it in the future? | Perceived usefulness |
| **11** | Where in your medical career would you be interested in using LLMs more? | Attitude towards use |
| **12** | If you have used LLMs during your medical education, how has this changed your performance during examinations and assessments? | Actual system use |
| **13** | Are there other type of AI which you think would remove the advantage of using LLMs or other things similar to LLMs you have used? | Attitude towards use |
| **14** | Would you be open to learning more about how to use LLMs to further your learning in the future? If yes/no – why? | Attitude towards use |
| **15** | How does LLMs compare to your current methods of learning? | Perceived usefulness |
| **16** | How do you think LLMs could be applied in the clinical world? | Perceived usefulness |
| **17** | Do you feel as time goes on and LLMs is used more, your examinations should be altered to accommodate for this, and if so how? |  |
| **18** | How do you think the value will change over the next three-five years? |  |
| **19** | Do you have any further questions or points to make which we may not have covered? |  |

**Codebook**

**Domain 1: System Use**

| **Sub-theme** | **Definition** |
| --- | --- |
| Topic summarisation | Use of LLMs to obtain concise explanations or summaries for medical/scientific topics. |
| Google/search engine replacement | LLMs as substitutes for traditional search engines for rapid question answering. |
| Quiz/exam question creation | Generating practice questions for study or self-testing using LLMs. |
| History-taking roleplay | Using LLMs to simulate clinical scenarios or patient interviews. |
| Email/admin drafting | Using LLMs for administrative writing tasks. |
| Article/research summary | Summarising research papers or guidelines with LLM assistance. |

**Domain 2: Perceived Usefulness**

| **Sub-theme** | **Definition** |
| --- | --- |
| Efficiency and time-saving | LLMs streamline access to information, reducing time taken for lengthy resource search or review. |
| Clarifying difficult concepts | LLMs help make difficult or complex concepts easier to understand. |
| Mnemonic/flashcard generation | Creation of study aids, such as mnemonics or flashcards |
| Change in educational assessment needs | LLMs can be integrated into regular revision or clinical routines. |

**Domain 3: Perceived Ease of Use**

| **Sub-theme** | **Definition** |
| --- | --- |
| Device flexibility | LLMs are accessible on various devices and used in differing environments. |
| Technical simplicity | Minimal technical expertise required. Users can just type a prompt and receive an answer. |
| Low learning curve | Students did not need formal teaching to start using LLMs. |

**Domain 4: Attitudes Towards use**

| **Sub-theme** | **Definition** |
| --- | --- |
| Difficulty trusting without prior knowledge | Students are less likely to trust LLM outputs for unfamiliar topics. |
| Hallucination/inaccurate answers | LLMs may generate plausible but incorrect or fabricated content ("hallucinations"). |
| Generating fake references/non-existent references | LLMs sometimes create references or links that do not exist or do not support claims. |
| Overreliance or dependency | Frequent use leads to risk of reduced skills in problem-solving or critical thinking. |
| Out-of-context output | Sometimes information does not match user intent or requires further prompting. |
| Privacy and data concerns | Hesitation to use LLMs for sensitive tasks due to privacy, copyright, or confidentiality. |
| Lack of awareness of LLM features | Students often do not know the full range of LLM capabilities. |
| Preference for official or older resources | Many students prefer traditional notes, textbooks, or guidelines for accuracy and reliability. |
| Inadequate for guideline/recommendation queries | Students are unlikely to use LLMs for management/treatment guidance. |
| Peer learning/social facilitation | Peer demonstration facilitates adoption and exploration of functions. |

**Recruitment Poster**
